# Prevention of the development of psychological distress following a motor vehicle crash: study protocol for a randomized controlled trial

**DOI:** 10.1186/s13063-016-1455-5

**Published:** 2016-07-16

**Authors:** Rebecca Guest, Yvonne Tran, Bamini Gopinath, Ian D. Cameron, Ashley Craig

**Affiliations:** John Walsh Centre for Rehabilitation Research, Kolling Institute for Medical Research, Sydney Medical School-Northern, The University of Sydney, Corner Reserve Road and First Avenue, St Leonards, NSW 2065 Australia

**Keywords:** Cognitive behaviour therapy, Motor vehicle crash, Prevention, Psychological distress, Psychological intervention

## Abstract

**Background:**

It is estimated that up to 50 % of motor vehicle crash survivors develop significant psychological distress, such as depressive mood and anxiety, within 6 months of the crash. Associated impacts include loss of employment, delayed return to work, financial and familial stress, and increased medical and compensation costs. The major aim of this research is to investigate the efficacy of interventions for preventing the development of psychological distress following a motor vehicle crash. The efficacy of two brief interventions will be examined: a cognitive behaviour therapy (CBT) programme, targeting mood and anxiety, and a lifestyle programme, targeting sleep, diet and physical activity.

**Methods/design:**

This is a randomized, controlled multisite study. Participants include at least 180 adults injured in a motor vehicle crash who have entered a compensation process. Research will compare outcomes in three groups randomly assigned to: one group of 60 adults, who receive a brief email-delivered CBT programme, with one session every 2 weeks for 10 weeks and telephone contact every 2 weeks; a second group of 60 adults, who receive a brief email-delivered lifestyle intervention involving one session every 2 weeks for 10 weeks with telephone contact; and an active waiting-list control group of 60 adults who are provided claims processing-related reading material along with telephone contact every 2 weeks for 10 weeks. Participants will be recruited within 12 weeks of the motor vehicle crash, and will be comprehensively assessed before and after treatment, and 6 and 12 months post-injury. Assuming an *α* probability level of 0.05 and a power of 80 %, at least 180 participants will be recruited. The primary outcome measure is the presence and severity of psychological distress or disorder. Secondary outcome measures include assessment of self-efficacy, resilience employment status, social activity and support, lifestyle and physical health factors, along with process outcome measures of treatment acceptability, feasibility and generalizability.

**Discussion:**

This study will determine whether brief email-delivered interventions distributed soon after the injury and entry into the claims process can be effective in preventing the development of psychological distress.

**Trial registration:**

ANZCTR, ACTRN12615000326594. Registered on 9 April 2015.

**Electronic supplementary material:**

The online version of this article (doi:10.1186/s13063-016-1455-5) contains supplementary material, which is available to authorized users.

## Background

Psychological distress is a common problem for those who have sustained a motor vehicle crash. For those who have entered the compensation system, the level of distress appears to be even more elevated [1]. Pain, injury, impairment, loss of income, trauma, depression and anxiety are common outcomes for survivors of motor vehicle crashes, and in an environment where coping capacity is already challenged, motor vehicle crash survivors are at risk of developing severe psychological disorders, such as major depressive disorder and post-traumatic stress disorder [1]. Such an outcome for motor vehicle crash survivors entering compensation systems is understandable, given the multiple post-injury stressors that can have an impact. Examples of possible stressors include continual reminders of crash-related trauma, when providing oral or written detailed accounts of the accident to insurance organizations, medical experts and legal representatives, reduced social participation and relationship conflicts [1]. It is estimated that up to 50 % of motor vehicle crash survivors will develop elevated psychological distress within 6 months of a crash [1], suggesting that addressing the risks associated with psychological distress early in the compensation process will be of benefit to at-risk people, as well as their employment capacities, families, employers and insurance companies [2].

Interventions that have potential for preventing the development of psychological distress and thus facilitate recovery following a motor vehicle crash are worthy of investigation. For example, motor vehicle crash survivors who file compensation claims could be educated in distress management early in the compensatory process, with the goal of enhancing psychological, social and behavioural skills. Alternatively, lifestyle programmes that improve sleep, diet and exercise might also better position survivors, improving their resilience and coping skills [3–5].

Cognitive behaviour therapy (CBT) is acknowledged as an efficacious treatment [1, 6], further evidenced by its support from government-sponsored initiatives [1]. Strategies used in CBT are pragmatic and effective, with easy-to-learn skills, such as problem solving, anxiety and mood management, in addition to psycho-education about stress and mental health. The efficacy of CBT has been established for mental health disorders, including depression, post-traumatic stress disorder and anxiety, and its efficacy has been demonstrated across a variety of clinical research studies [1, 5, 7–9]. Therefore, CBT should benefit those who have experienced a motor vehicle crash, and who are engaged in the compensation system.

Additionally healthy lifestyle programmes have also been demonstrated to have positive health outcomes in various populations and communities [4, 5, 10]. While there are a limited number of studies that have investigated the efficacy of healthy lifestyle strategies for mental health, current research suggests that positive mental health is independently associated with lifestyle features, such as regular physical activity [3]. Conversely, reduced physical activity has been found to be associated with health risks, such as coronary heart disease, Type II diabetes, some cancers, obesity, hypertension, depression and anxiety disorder [11]. Physical fitness, indicated by a lower heart rate, was found to be associated with smaller inflammatory responses to mental stress as well as great parasympathetic control (e.g. through increased vagus nerve activity or lower neural arousal reactivity to stress) [3]. These factors are known protective factors of mental health disorders, such as depression [12]. Similarly, poor sleep quality has been associated with a variety of health behaviours, impairment and quality of life [13]. A systematic review of healthy lifestyle programmes delivered by internet [4] revealed that such factors as peer and professional support, regular email or phone contact and education were related to increased adherence and better outcomes in participants.

The internet has provided a new format for delivering CBT or healthy lifestyle interventions, by way of an online bibliotherapy with therapist contact [14]. Here, the client is exposed to the same components as with traditional face-to-face treatment; however, the psycho-educational material and instructions for behavioural change are delivered using an internet-based treatment platform [9]. A recent systematic review identified that an online-delivered CBT programme was cost-effective compared with face-to-face CBT, based on improvements in symptoms and reduction of time and resource demands on clinicians [9]. However, recent research has suggested that online-delivered CBT designed to treat depression is no more effective than usual face-to-face care [15]. Here, the low user engagement might have reflected the fact that direction to a website is perceived as impersonal and requires a degree of motivation perhaps unavailable to a clinically depressed person [15, 16]. For this trial, the intervention will be delivered by email and is expected to have similar efficacy to an online programme [9], with the advantage of substantially reduced web-based developmental costs, as well as a more personally delivered CBT treatment via email in addition to telephone support specific to CBT strategies of gentle guidance and support of specific skills and strategies.

To our knowledge, there have only been six randomized controlled trials investigating the prevention of psychological distress following a motor vehicle crash. All six have involved relatively small samples [17–22]. Of these, only two specifically focused on compensatory claimants [17, 18], and these both had non-significant results. However, other studies have reported significant results in reducing symptoms of psychological distress in people with elevated distress levels following a motor vehicle crash using CBT. For example, CBT techniques involving exploration of thoughts and feelings and *in-vivo* exposure [23, 24] and cognitive restructuring [25] have either contained or reduced symptoms of anxiety, depression and post-traumatic stress disorder for motor vehicle crash survivors. These promising results suggest that a randomized controlled trial involving interventions such as CBT, and designed to prevent development of psychological distress, employing a sample size with sufficient power to detect a true finding, is warranted. The uniqueness of this trial is that it will investigate the efficacy of interventions designed to prevent development of psychological distress for people entering the compensation system following a motor vehicle crash. Participants will be included, regardless of whether or not they have symptoms of psychological morbidity.

### Aims of the study

The major aim of the study is to evaluate the effectiveness of an intervention designed to prevent development of psychological distress following a motor vehicle crash and delivered within 12 weeks of the crash. A secondary aim is to achieve a relatively high rate of adherence and participant satisfaction with email-delivered programmes, consistent with other studies of online-delivered CBT treatments [26]. Primarily, the treatment interventions aim to prevent the development of psychological distress, along with producing clinically and statistically significant improvements in health outcomes, such as reduced anxiety and depressive mood, increased physical activity, improved sleep, improved quality of life and disability ratings, reduced pain severity, and improved return to pre-injury employment and daily functioning in the medium term after the programme. Table 1 shows the flow of the trial’s recruitment, intervention and assessments.

**Table 1:** SPIRIT flow chart for recruitment, treatment and assessments

	Study period							
	Enrolment	Randomization	Treatment (5 fortnightly emailed programmes with phone calls)					6 and 12 month follow-up sessions
Time point	−*t*_1_	0	T1	T2	T3	T4	T5	
Recruitment:								
Eligibility screen	×							
Informed consent	×							
Randomization		×						
Interventions:								
Cognitive behaviour therapy			×	×	×	×	×	
Lifestyle			×	×	×	×	×	
Waiting-list control			×	×	×	×	×	
Assessments:								
1. Baseline (demographics, lifestyle, health)		×						
2. Post-treatment lifestyle, health, process outcomes							×	
3. 6-month lifestyle, health								×
4. 12-month lifestyle, health								×

## Methods/design

This study is designed as a three-arm randomized controlled trial with two active interventions and one active waiting-list control. The first is a brief email-delivered CBT treatment, consisting of five modules delivered fortnightly. The second is a brief email-delivered lifestyle treatment, also consisting of five modules and delivered fortnightly. Both groups will also receive weekly telephone contact to encourage their engagement with their programmes. The active waiting-list control group will receive reading material about the claims process, and will also receive fortnightly follow-up phone calls; however, these calls will only involve brief discussions of their progress in the compensation scheme in addition to confirming that they are receiving their reading material. There will be four measurement points: baseline (within 12 weeks of the motor vehicle crash), post-treatment (within 2 weeks of cessation of the interventions), and 6-month and 12 month follow-up sessions for the treatment groups (from the time of entry into the study) with the final two assessments also including participant satisfaction and acceptability questionnaires. If participants in the waiting-list control group do not want to receive either of the intervention programmes, they will also be assessed at 12 months.

### Recruitment, inclusion and exclusion criteria

Recruitment involves an opt-in two-step process for adult motor vehicle crash survivors who have lodged a compensation claim with one of the multisite insurance companies. Insurance companies consisted of one major Compulsory Third Party insurance company in New South Wales and one in Victoria, Australia. Step 1 involves screening by the claimants’ insurance case manager for interest in participating in the research. Step 2 involves the researcher telephoning the potential participant to discuss the research further, followed by emailing information sheets and consent forms to those people who have indicated willingness to participate.

#### Inclusion criteria

1. Adult (aged 18+) survivors of a motor vehicle crash who have lodged a compensation claim within 3 months of their accident
2. English speaking

#### Exclusion criteria

Catastrophic or complex injuries such as

- spinal cord injury
- amputation
- blindness
- multiple fractures
- internal damage requiring extended hospitalization
- severe traumatic brain injury

### Sample size

**Fig. 1:**
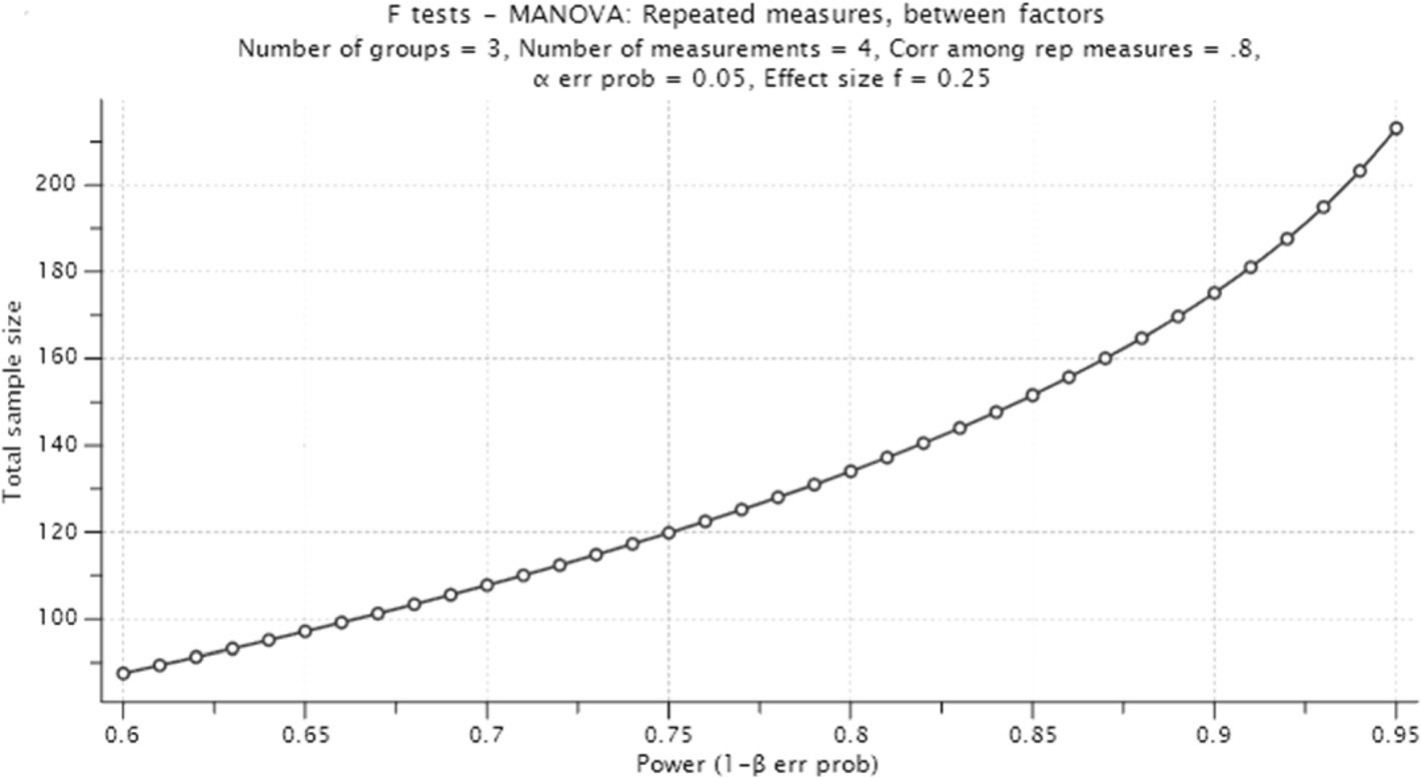
Power analysis

Based on prior results of CBT and healthy lifestyle interventions [1, 4, 5], the two interventions are assumed to have at least a small to moderate mean effect size of 0.25 (Cohen’s *d*) when compared with the control group. Assuming an α probability of 0.05 and a power of 80 % for a three-group comparison analysis with four measures over time, a sample of 135 participants must be recruited to detect a true difference (see Fig. 1). It is therefore proposed to recruit at least 180 adult participants, randomly assigned to one of the three groups each with at least 60 participants per group. This is to accommodate for the loss to follow-up of approximately 25 % of participants, based on similar research [27].

### Type of trial

This study is designed as a multisite, randomized controlled clinical intervention trial.

### Randomization and blinding

Randomization will be determined by the database analyst, who will develop a macro in Microsoft Excel. The macro will be programmed to generate random combinations from six possible permutations (123, 132, 213, 231, 321, 312). Participants will be randomized according to group (for example: 1 = CBT; 2 = lifestyle; 3 = control) based on their entry into the trial and the corresponding permutation. The random group assignment will be kept separately from all online assessments in a pass-coded programme under password security. Participants will not be advised of their randomization, and the database analyst, statistician and study co-ordinator will all be blind to both randomization and group name and number.

### Interventions

All participants will be randomly assigned to one of three groups: (i) CBT, (ii) lifestyle or (iii) waiting-list control. For the two treatment groups, participants are delivered fortnightly modules presented in Microsoft PowerPoint presentation with homework worksheets and templates for self-monitoring and practising learnt skills. Emails are brief with two or three paragraphs containing three or four concise sentences as well as a brief case study followed by a weekly summary. At the beginning of every other week, participants are telephoned for a maximum 10 min conversation aimed at (i) encouraging the learning and practising of skills from the previous week, (ii) ‘normalizing’ the challenges of learning new skills, (iii) emphasizing that symptom reduction requires gentle but consistent practice of the skills over time, and (iv) reminding participants about unread materials. The control group is also emailed fortnightly; however, this is restricted to reading information relating to the claims process, and phone calls are aimed at ensuring they are receiving and reading this information. The control group will be offered the programme of their choice, or the programme analyzed as more efficacious after their 6-month follow-up assessment. The contents of each module for each group (original version, March 2015) are presented in Table 2.

**Table 2:** Contents of modules for cognitive behaviour therapy, lifestyle and waiting-list control groups

	Cognitive behaviour therapy	Lifestyle	Control
1	Overview	Overview	Reading: ‘A guide for people injured in a motor vehicle crash’
2	Mood and slow breathing skills, self-monitoring	Goal setting and life pacing	Reading: ‘Overview of the claims process’
3	The art of distraction, applying distraction	Self-monitoring and changing unhelpful behaviours	Reading: ‘Obligations of the insurer’
4	Stress and helpful thinking, evidenced-based thinking	Sleep hygiene and sleep self-monitoring	Reading: ‘Obligations of the claimant’
5	Problem solving and conclusions, self-mastery, changeable versus unchangeable problems	Improving well-being through diet and physical exercise, social participation, daily activity schedule	Reading: ‘Finalizing a claim’

### Outcome measures

#### Primary and secondary measures

Table 3 shows the primary and secondary outcome measures. The primary outcome measure is the presence and severity of psychological distress or disorder across four time points assessed by a mixture of psychometric mental health measures and diagnostic interviews based on criteria of the fifth edition of the *Diagnostic and Statistical Manual of Mental Disorders* for depression, anxiety and post-traumatic stress disorder. Secondary outcome measures will include motor vehicle crash injury history, employment, social life, resilience and self-efficacy, as well as indicators of physical health and lifestyle.

**Table 3:** Primary and secondary outcome measures acrossbaseline, post-treatment and follow-up

Measure	Primary	Secondary	Baseline	3 months	6 and 12 months
Demographics					
Sex, age, weight, height		×	×		
Marital status		×	×		
Highest level of education		×	×		
Motor vehicle crash history					
Motor vehicle crash date		×	×		
Driver or passenger		×	×		
Perception of danger during motor vehicle crash		×	×		
Hospital admission: yes or no		×	×		
Days spent in hospital		×	×		
Involvement of lawyer		×		×	
Referral to psychologist or psychiatrist		×		×	
Satisfaction with claims process: 3-point Likert scale		×			×
Claim finalization: yes or no		×			×
Employment					
Pre-motor vehicle crash employment: part-time, full-time, pensioner		×	×		
Modified hours since motor vehicle crash: yes or no		×	×	×	×
Returned to work: yes or no		×	×	×	×
Expectation of return to work: 11-point Likert scale		×	×	×	×
Reason for non-return to work		×	×	×	×
Number of days after motor vehicle crash for return to work		×	×	×	×
Social life					
Returned to activities after motor vehicle crash: yes or no		×	×	×	×
Expectation of return to pre-motor vehicle crash activities: 11-point Likert scale		×	×	×	×
Pre-motor vehicle crash social activity quality		×	×	×	×
Satisfaction of pre-motor vehicle crash social supports: 11-point Likert scale		×	×	×	×
Satisfaction of pre-motor vehicle crash social engagements		×	×	×	×
Expectation of return to pre-motor vehicle crash activities: 11-point Likert scale		×	×	×	×
Pre-motor vehicle crash relationship quality: 5-point Likert scale		×	×	×	×
Lifestyle habits					
Alcohol: Alcohol Use Disorders Identification Test	×		×	×	×
Smoker: yes, no, amount per day		×	×	×	×
Health					
Health Status: SF-12		×	×	×	×
Psychology or psychiatric history: yes or no		×	×	×	×
Mood medication history: yes or no		×	×	×	×
Motor vehicle crash self-blame: 11-point Likert scale		×	×	×	×
Motor vehicle crash others-blame: 11-point Likert scale		×	×	×	×
Motor vehicle crash: perception of risk of own death: 11-point Likert scale		×	×	×	×
Coping capacity: The COPE		×	×	×	×
Pain: 11-point Likert scale		×	×	×	×
Pain catastrophizing: Pain Catastrophizing Scale		×	×	×	×
Impact of motor vehicle crash: Impact of Event Scale		×	×	×	×
Mood: Depression Anxiety Stress Scale 21	×		×	×	×
Resilience: Connor–Davidson Resilience Scale		×	×	×	×
Mood states: Profile of Mood States	×		×	×	×
*Diagnostic and Statistical Manual of Mental Disorders* Mini Plus Scores	×		×	×	×
Depression	×		×	×	×
Anxiety	×		×	×	×
Panic	×		×	×	×
Specific phobias	×		×	×	×
Post-traumatic stress disorder	×		×	×	×
Suicidal thoughts: yes or no	×		×	×	×

#### Process measures

Treatment acceptability will be assessed by determining satisfaction with the intervention based on telephone calls to participants during treatment, and by asking four questions after treatment:

1. Overall, how satisfied were you with the programme?
2. How satisfied were you with the modules and module summaries?
3. Would you feel confident in recommending this treatment to a friend?
4. Was it worth your time doing the programme?

Participants will respond to the first two questions using a five-point Likert scale, which will range from ‘very satisfied’ to ‘very dissatisfied’ and the second two questions with a simple ‘yes’ or ‘no’ response. These questions have been used in previous research to examine the acceptability of internet-delivered CBT and other similar low-intensity treatments amongst consumers with a range of different conditions and across a range of different age groups [28–30]. Treatment feasibility will be broadly assessed based on the preliminary outcomes achieved combined with the ease with which clients could be supported through the programme by the insurance regulatory agencies. Generalizability of the treatment will be assessed with reference to the percentage of people who enter the study compared with the total number to whom participation is offered, and the follow-up rate achieved.

#### Safety measures and endpoint

This study is being conducted by a clinical psychologist highly experienced in the assessment and treatment of mental health disorders, as part of a doctoral study. The occurrence of adverse events, such as severe episodes of anxiety, panic, depression of risk of self-harm during the interventions, are considered safety endpoint measures of this trial. The regular contact by the clinical psychologist will identify whether the safety endpoint measure is required by any participant and appropriate action as per accepted mental health clinical guidelines will be instigated.

### Hypothesis and data analysis

The primary hypotheses assert that brief interventions (CBT and healthy lifestyle) will prevent the development of psychological distress by improving psychological coping skills and healthy behaviours. A further hypothesis is that the intervention groups will show improved resilience and quality of life compared with the control group; however, we are not hypothesizing a significant difference between the treatment groups themselves.

The effectiveness of the interventions will be determined by conducting multivariate analyses of variance with repeated measures for revealing differences within and between groups, as well as reporting effect size changes. Potential confounding factors, such as pre-injury health and education, will be adjusted for in the analysis. Correlation analyses and multiple regression will also be used to analyse predictors of psychological distress over time. The intervention satisfaction questionnaire will be analyzed to assess participant satisfaction of the overall treatment approach. Clinically significant improvements or recovery will be determined by identifying the proportion of participants who demonstrate a significant reduction in their symptoms.

An interim analysis will re-evaluate the assumptions made for the study’s sample size calculation, with re-estimation made if required, unless the trial needs to be stopped as a result of significant and consistent indications of elevated psychological distress associated with the interventions, according to the safety measures and endpoint.

### Trial organization, registration and ethical aspects

To ensure data integrity the database analyst remains blind to participant allocation details. Data are to be saved in three different locations under tight security with pass-code access only. An additional SPIRIT checklist file shows how the recommendations of a clinical trial protocol have been addressed (see Additional file 1).

## Discussion

This study will allow us to determine whether development of psychological distress and psychological disorders, such as major depressive disorder and post-traumatic stress disorder, can be prevented for motor vehicle crash survivors using brief email-delivered intervention programmes aimed at reducing psychological distress, and improving quality of life, coping and resilience. If the result is positive, and the intervention is acceptable to clients and efficient to administer, it could be implemented by the insurance companies’ regulatory agency as part of standard protocol to all motor vehicle crash survivors. Such a protocol would have the potential to reduce the psychological and economic burdens of motor vehicle crashes with benefits for at-risk people and their families, as well as their employers, with more efficient return to work timeframes, while reducing costs for insurance companies. The results of the study would enable other national and international regulatory agencies to implement a similar preventive intervention strategy.

## Trial status

Two sources of recruitment (national companies with local branches) have been initiated and are actively providing potential participants. The first participant was recruited on 8^th^ August 2015. The expected recruitment period will last until July 2017.

## Abbreviations

ANZCTR, Australian New Zealand Clinical Trials Registry; CBT, cognitive behaviour therapy; SPIRIT, Standard Protocol Items: Recommendations for Interventional Trials

## Additional file

Additional file 1: SPIRIT 2013 checklist: recommended items to address in a clinical trial protocol. (PDF 131 kb)
